# Integrated vector management: a critical strategy for combating vector-borne diseases in South Sudan

**DOI:** 10.1186/1475-2875-12-369

**Published:** 2013-10-25

**Authors:** Emmanuel Chanda, John M Govere, Michael B Macdonald, Richard L Lako, Ubydul Haque, Samson P Baba, Abraham Mnzava

**Affiliations:** 1 Population Services International, Juba, South Sudan; 2 Ministry of Health, Juba, South Sudan; 3 Vector Control Specialist/Consultant, Nelspruit, Mpumalanga, South Africa; 4 Global Malaria Programme, World Health Organization, Geneva, Switzerland; 5 W. Harry Feinstone Department of Molecular Microbiology & Immunology, Johns Hopkins Bloomberg School of Public Health, Baltimore, MD 21205, USA

**Keywords:** Malaria vector control, Integrated vector management, Policy and strategy, Community involvement, Intersectoral collaboration, Capacity building, Monitoring and evaluation, South Sudan

## Abstract

**Background:**

Integrated vector management (IVM) based vector control is encouraged by the World Health Organization (WHO). However, operational experience with the IVM strategy has mostly come from countries with relatively well-established health systems and with malaria control focused programmes. Little is known about deployment of IVM for combating multiple vector-borne diseases in post-emergency settings, where delivery structures are less developed or absent. This manuscript reports on the feasibility of operational IVM for combating vector-borne diseases in South Sudan.

**Case description:**

A methodical review of published and unpublished documents on vector-borne diseases for South Sudan was conducted via systematic literature search of online electronic databases, Google Scholar, PubMed and WHO, using a combination of search terms. Additional, non-peer reviewed literature was examined for information related to the subject.

**Discussion:**

South Sudan is among the heartlands of vector-borne diseases in the world, characterized by enormous infrastructure, human and financial resource constraints and a weak health system against an increasing number of refugees, returnees and internally displaced people. The presence of a multiplicity of vector-borne diseases in this post-conflict situation presents a unique opportunity to explore the potential of a rational IVM strategy for multiple disease control and optimize limited resource utilization, while maximizing the benefits and providing a model for countries in a similar situation.

**Conclusion:**

The potential of integrating vector-borne disease control is enormous in South Sudan. However, strengthened coordination, intersectoral collaboration and institutional and technical capacity for entomological monitoring and evaluation, including enforcement of appropriate legislation are crucial.

## Background

Vector-borne diseases account for about 17% of the estimated global burden of infectious diseases [1] and exert an enormous toll on the continent of Africa [2]. They result in loss of productivity, school absenteeism, and aggravation of poverty, high costs for health care and a burden on public health services [3]. Past and current efforts at controlling most vector-borne diseases have relied solely on disease management [4]. As neither effective medication nor vaccines are available for some of these diseases, vector control remains pivotal. Vector control is crucial to reduce the extent to which drugs are needed to treat the diseases, as the parasite can become resistant, or the drugs are often unaffordable for those most affected [5]. While vector control has a long-standing, proven record of preventing, reducing and eliminating vector-borne diseases [1], when available, vector control programmes are mostly vertical, even when and where targeted communities are afflicted by multiple diseases [6]. The need to integrate efforts and optimize the use of limited available human and financial resources is evident.

In response to the challenges, the World Health Organization’s (WHO) integrated vector management (IVM) strategy, a rational decision-making process to optimize use of resources, was established as a pivotal platform for combating these often chronic and debilitating diseases [7]. The strategy is based on the premise that effective control is not the sole preserve of the health sector but of various public and private agencies, including communities. Salient attributes of IVM include: methods based on knowledge of factors influencing local vector biology; disease transmission and morbidity; use of a range of interventions, often in combination and synergistically; collaboration within the health sector and with other public and private sectors that impact on vectors; engagement of local communities and other stakeholders; a public health regulatory and legislative framework. The IVM strategy has five key elements: advocacy; social mobilization and legislation; collaboration within the health sector and with other sectors; integrated approach; evidence-based decision-making; and capacity building.

An IVM-based process should be cost-effective, guided by operational research and subject to routine monitoring and evaluation of impact on vector populations and disease transmission, including development of an infrastructure, financial resources and adequate human resources to manage and implement integrated vector control programmes at national and local levels [7]. As such, control programmes are encouraged to adopt and implement the approach. Since the strategy was introduced [7], and through WHO’s close collaboration with member states, 68 vector-borne disease-endemic countries have established national policies for IVM [8]. To help member states re-orient to IVM and be able to meet the growing challenges amidst dwindling public-sector human and financial resources, the WHO provides technical assistance to facilitate the implementation processes [3], guidance on policy-making [6], structure for training [9] and monitoring and evaluation [10]. However, very few programmes have harnessed this approach for mounting a rational, effective and integrated operational offensive to combat multiple vector-borne diseases.

Upon the signing of the Comprehensive Peace Agreement in 2005, South Sudan has been characterized by enormous infrastructure, human and financial resource constraints and a weak health system against a huge burden of vector-borne diseases [11]. The country is thought to be one of the heartlands of vector-borne diseases in the world [12]. Communities are commonly at risk from more than one vector-borne disease. Accordingly, the Ministry of Health (MoH) has adopted the IVM strategy to strengthen the control and prevention of these diseases [13]. While this situation invariably poses a serious challenge to socio-economic development in this post-emergency setting, the presence of a multiplicity of vector-borne diseases presents a unique opportunity to explore the potential of IVM and optimize the utilization of the limited available resources, while maximizing the benefits.

A growing body of evidence demonstrates that vector control significantly reduces illness, social exclusion and mortality [14] and will thus contribute directly to the attainment of several Millennium Development Goals [15]. Therefore, this paper stresses the need for rational decision-making and sustained support for vector control in South Sudan and also serves as an archetype for other similar post-conflict environments.

### Case description

A methodical review of published and unpublished documents on vector-borne diseases for South Sudan was conducted via systematic literature search of online electronic databases: Google Scholar [16], PubMed [17] and WHO [18] using a combination of search terms: 1) malaria AND IVM; 2) NTDs AND IVM; 3) NTDs AND vector control; 4) Southern Sudan OR South Sudan; 1) and 4); 2) and 4); and, 3) and 4); vector control, epidemiology, malaria, human African trypanosomiasis (sleeping sickness), visceral leishmaniasis (kala-azar), onchocerciasis (river blindness), lymphatic filariasis, dracunculiasis (Guinea worm), schistosomiasis (Bilharzia), loiasis, dengue and yellow fever. Additional non-peer reviewed literature was examined for information related to the subject.

### Study area

**Figure 1:**
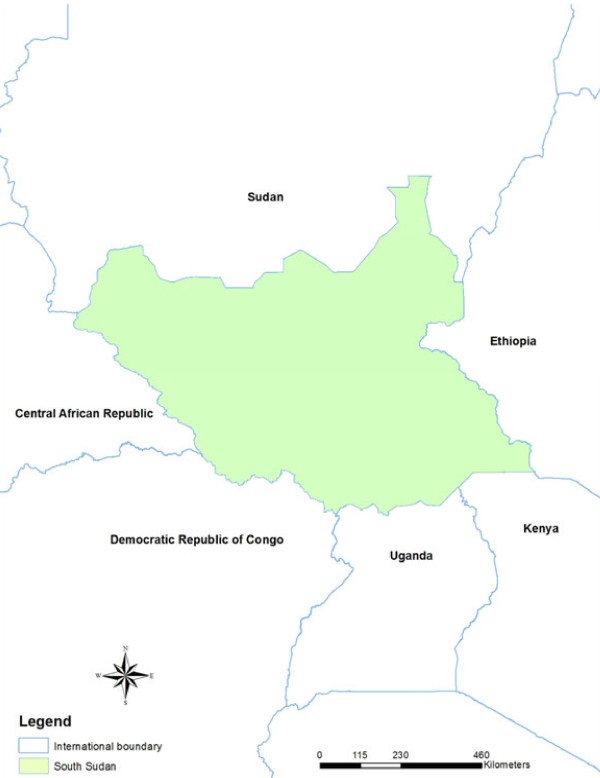
The map of South Sudan.

South Sudan covers 650,000 sq km of land between 8° and 18° south latitude and between 20° and 35° east longitude with a population of 8.3 million and almost 900,000 refugees, returnees and internally displaced persons [19]. The country is landlocked in East Africa bordering the Democratic Republic of Congo in the southwest, Uganda and Kenya in the south, Central African Republic in the west, Sudan in the north and Ethiopia in the east [Figure 1]. The country’s vegetation is dominated by savannah with only minor mountains. The climate is tropical with average temperatures ranging between 20 and 37°C and relative humidity between 26 and 88%. Annual rainfall ranges between 1,000 mm in the south and 400 mm in the northern parts. Similarly, the duration of the rainy season is longest in the south (seven to eight months) and reduces towards the northern part (five to six months). Population growth rate is estimated to be 2.85% with more than 90% living on less than US$1 per day and the poverty rate of between 40 and 50% [19]. Agriculture is the main source of income for more than 85% of the population. All-cause infant mortality rate is estimated at 150/1,000 live births, under-five mortality rate 250/1,000 live births and maternal mortality ratio 1,700/100,000 live births [11].

### The rationale for integrated vector management

Although vector control has a proven record of reducing the burden of vector-borne diseases [7], its benefits are far from being realized because of: 1) the scarcity of skills to manage and implement vector control; 2) the development of insecticide resistance in disease vectors; and, 3) minimal or lack of collaboration between infrastructure development programmes and the health sector [20]. WHO has developed a Global Strategic Framework that sets out the principles and approaches to IVM [7,21]. While IVM is an acknowledged approach for the prevention and control of vector-borne diseases [22], operational experience has, to date, been from countries with relatively well-established health systems [23,24]. Little is known about deployment in post-conflict settings, where delivery structures are lacking or less developed. Very little has been done to combat multiple vector-borne diseases. South Sudan provides a unique opportunity to develop an integrated programme providing a good chance for the generation and establishment of entomological and epidemiological baseline data for future tracking of performance of interventions.

### The scale of the vector-borne disease problem

South Sudan carries a disproportionate share of the regional and global burden of vector-borne diseases. Eleven per cent of the global burden due to vector-borne diseases is found in countries of the Eastern Mediterranean where only 8% of the global population lives. It is estimated that 4.8% of the global burden due to vector-borne diseases is contributed by South Sudan alone [22]. These statistics highlight the devastating impact of vector-borne disease on human health and socio-economic development of many communities. The world’s most predominant vector-borne diseases are endemic in South Sudan with varying extents of geographical spread [12] (Table 1). Most vector-borne diseases have received global attention and the World Health Assembly (WHA) has passed resolutions (Table 2), but the problem remains enormous in South Sudan with unequal levels of control in place. Furthermore, rebuilding and rehabilitation efforts in post-conflict South Sudan have predisposed people from all walks of life to vector-borne diseases.

**Table 1:** The burden of vector-borne diseases endemic in South Sudan

**Disease**	**Causative agent**	**Vector**	**Distribution**	**Burden**	**Intervention**	**References**
Malaria	*Plasmodium falciparum P. vivax*	*Anopheles gambiae, An. arabiensis, An. funestus*	Countrywide	1.2 million cases in 2012	ITNs, IRS	[25]
Human African trypanosomiasis (sleeping sickness)	*Trypanosoma brucei gambiense T. b. rhodesiense*	*Glossina fuscipes G. tachinoides, G. pallidipes, G. morsitans*	Greater Equatoria Region, Jonglei state (Akobo County)	1-2 million people at risk	Introduce and maintain vector control (tsetse traps)	[26,27]
Visceral leishmaniasis (kala-azar)	*Leishmania donovani*	*Phlebotomus orientalis P. martini*	Unity, Jonglei, UN and EE	Cyclic (500 – 9,000 cases/year)	LLINs in highly endemic communities	[12,28]
Lymphatic filariasis (elephantiasis)	*Wuchereria bancrofti*	*Anopheles gambiae, An. arabiensis, An. funestus*	Mapping not completed, but probably all ten states	Unknown	Large-scale distribution of LLINs for vector control	[28]
Loiasis	*Loa loa*	*Chrysops silacea* and *C. dimidiata*	Equatoria region; predominantly WE	Unknown	Large-scale distribution of LLINs for vector control	[12]
Onchocerciasis (River blindness)	*Onchocerca volvulus*	*Simulium damnosum*	Hyperendemic in WBEG, NBEG, Warrap, Lakes, WE, CE and parts of EE; Parts of Unity; Jonglei and UN	4.1 million at risk	Larvicides	[12,28]
Dracunculiasis (Guinea worm)	*Dracunculus medinensis*	Chrysops	All states except WE and Unity	3,618 cases in 2008, by June 2009, 1,188 cases reported	Water filtration and treatment of water sources	[28,29]
Schistosomiasis (Bilharzia)	*Schistosoma haematobium S. mansoni*	*S. haematobium S. mansoni*	Probably Warrab, Lakes, Unity and UN	Unknown	Molluscicides	[12,28]

*Abbreviation* of States: *BN*, Blue Nile, *CE*, Central Equatoria, *EE*, Eastern Equatoria, *NBEG*, North Bahr el Ghazal, *UN*, Upper Nile, *WBEG*, Western Bahr el Ghazal, *WE*, Western Equatoria.

**Table 2:** The global burden of major vector-borne diseases and WHA resolutions for combating them

**Disease**	**DALYS (X 1000)**	**WHA resolution number**	**Title**	**Year**
Human African trypanosomiasis	3,700	WHA57.2	Control of human African trypanosomiasis	2004
Leishmaniasis	2,100	WHA60.13	Control of leishmaniasis	2007
Dracunculiasis		WHA64.16	Eradication of dracunculiasis	2011
Lymphatic filariasis	5,800	WHA50.29	Elimination of lymphatic filariasis as a public health problem	1997
Onchocerciasis	500	WHA62.1	Prevention of avoidable blindness and visual impairment	2009
Schistosomiasis	1,700–4,500	WHA54.19	Schistosomiasis and soil-transmitted helminth infections	2001
Malaria	45,000	WHA42.31	Control of disease vectors and pests	1989
		WHA50.13	Promotion of chemical safety, with special attention to persistent organic pollutants	1997
Dengue	700	WHA55.17	Prevention and control of dengue and dengue haemorrhagic fever	2002

### Status of IVM implementation

Recognizing the importance of vector-borne diseases in the Eastern Mediterranean Region (EMRO), member states endorsed IVM as a regional strategic approach through a WHO Regional Committee Resolution (EM/RC.52/R.6) in 2005 [30]. The WHO/EMRO region has demonstrated progress in translating the IVM strategy into action at national level [22]. In South Sudan, the WHO-led IVM strategy has been adopted as the main platform for the control and prevention of vector-borne diseases in accordance with the WHO framework for IVM implementation [13]. A strategic plan for IVM has been developed with the objective: “To implement IVM for the prevention of vector-borne diseases through the deployment of cost-effective and sustainable vector control interventions and strengthened intersectoral coordination, partnerships and community empowerment” [7]. However, the strategy has been minimally utilized for malaria vector control [31] due to a lack of requisite capacity for implementation and limited collaboration among stakeholders.

To improve IVM implementation, technical capacity at the national malaria control programme has been strengthened to support MOH undertake a comprehensive situation analysis to establish vector control needs, current capacities and any gaps that need to be addressed and to put in place appropriate policies, strategies, operational guidelines and tools for IVM. An entomological laboratory and IVM monitoring system to track key vector control indicators including the development of an evidence base to inform policy recommendations is being established. Efforts to establish functional multi-disciplinary coordination mechanisms for IVM and to advocate for appropriate vector control interventions among key stakeholders have been embarked upon.

### Opportunities for effective IVM implementation

Following the peace agreement after 30 years of conflict, there is a high political commitment to addressing communicable diseases, encompassing the availability of a national health policy document and a wide range of partners currently supporting various vector control activities; availability of the IVM strategic plan; good relationship with development partners and a high commitment of line ministries to jointly support vector control with the MoH. This provides an opportunity for coordinated management of various diseases and use of different vector control tools to control specific diseases based on their sympatric occurrences. The IVM approach will strengthen the efforts by preventive chemotherapy for vector-borne neglected tropical diseases [12], improve management of insecticides and effective mitigation of potential negative health and environmental impacts, and provide a sound basis for management of insecticide resistance in disease vectors. It will enhance intersectoral accountability, leading to responsible actions among a wide range of stakeholders and provide a framework to sustain and maximize the impact of vector control interventions with optimal utilization of available resources [7].

Implementation of IVM requires explicit understanding of spatio-temporal patterns of vector-borne diseases and influencing factors that need technology that incorporates all the necessary information in an efficient manner. Geographic information system (GIS) developed from computerized cartography design systems represents an important tool for this purpose. Both GIS and associated technologies such as environmental remote sensing (RS) to characterize local conditions and also Global Positioning System (GPS) to locate ecologic features represent critical enabling technologies for epidemiologists, vector biologists and malariologists to characterize various environmental conditions and link them with vector-borne disease data in both space and time. Remotely sensed data provides important, up to date information about the environment (natural or social) in vector-borne disease control operational areas at a resolution that can rarely be obtained on the ground. Such data can be processed to provide potentially relevant information about environmental conditions critical for IVM implementation.

### Challenges for establishing a viable IVM strategy

Mounting a formidable offensive against an array of chronic and debilitating vector-borne diseases is highly compromised by various reasons: environmental, socio-cultural, socio-economic, technical and programmatic; a weak health system; limited access to health services; lack of accurate entomological and epidemiological data to guide vector control planning and response; pesticide management and the threat of insecticide resistance development; weak planning and coordination amongst disease control programmes; a severely constrained skilled human resource base to drive the vector control agenda forward. South Sudan’s public health system and other services remain devastated from the legacy of violence and instability. The majority of the country is rural and waterlogged with a minimal or non-existent road network. Population increase, returnees, internally displaced and nomadic behaviour of people precludes effective deployment of interventions. Utilization of long-lasting insecticidal bed nets (LLINs) is compromised by their abuse/misuse and most houses are not amenable to indoor residual spraying (IRS). This is further aggravated by extremely minimal intersectoral collaboration among public and private sectors including community empowerment, involvement and participation. Integration and coordination among vector-borne disease control programmes remain negligible. Appropriate requisite regulatory and legislative framework for public health is also non-existent. There is minimal evidence-based decision-making to provide technical guidance to policymakers and programme managers. Limited operational research and spatio-temporal mapping of vector-borne diseases across the country are also among the major challenges.

Effective vector-borne disease control demands for diligent entomological capacity, as such, there is need for medical entomologists and vector control specialists to spearhead IVM. South Sudan experiences a serious lack of vector control capacity at national, state and county levels. The major limitations for capacity-building for IVM are lack of essential physical infrastructure (insectaries, laboratories and equipment), financial resources, and technical resources (qualified vector control human resources) to support entomological monitoring and evaluation of vector control interventions. The technical assistance provided by the very few resources that exist is extremely overstretched. Addressing deficiencies in all these areas of public health capacity would be necessary for the successful implementation of IVM in South Sudan. This would need strengthened collaboration with stakeholders including local and international academic and scientific institutions and line ministries (environment and agriculture) to facilitate for entomological human resource and infrastructural strengthening.

## Discussion

In response to the call by WHO for member states to implement the IVM strategy [7], most countries in sub-Saharan Africa are deploying the approach for malaria vector control [23,24]. Well-established IVM strategies with adherence to the five key attributes have demonstrated the enhanced impact of interventions and opened a window for leveraging additional resources [24]. Very few have harnessed the strategy for combating multiple vector-borne diseases. While the need for effective vector control in South Sudan is huge, the country provides a unique enabling environment for incorporating other diseases in the IVM approach to rationalize the use of limited available human and financial resources. Effective vector control will require operations research and implementation of new innovative tools, including improvements in geographical information systems and satellite imagery to enhance targeting of interventions. Unless all stakeholders recognize the significance of IVM and provide the resources and commitment to implement the strategy, vector-borne disease control will remain fragile in the country.

In South Sudan, limited human resource and supporting infrastructure pose great hindrance to effective IVM [11] resulting in deployment of unsuitable and poorly targeted vector control interventions with insufficient coverage and wastage of resources. Cooperation between development sectors and health sectors has been poor or non-existent. There is minimal awareness of preventive measures and surveillance for trypanosomiasis [26]. Vector control remains a major challenge for schistosomiasis as control revolves largely around the use of drugs. Insecticide usage in agriculture and poor management in public health can result in development of insecticide resistance in disease vectors and compromise vector control [32]. The WHO Pesticide Evaluation Scheme (WHOPES) advises member states on the judicious and low-risk use of pesticides and their sound management [33]. Vector control needs new environmentally friendly pesticides and resistance management strategies to address the growing challenge of insecticide resistance [32]. IVM has been recognized as the most practical approach to sustainable vector control noting the limitations normally encountered with individual characteristic interventions [33]. Within the framework of the five key attributes of IVM, there is great potential for effectively overcoming the challenges that vector control is currently facing.

Evidence-based decision-making that allows for adaptation of strategies and interventions to local vector ecology, epidemiology and resources and the prevailing socio-economic conditions is critical [7]. Given the multiplicity of vector-borne diseases in South Sudan, including the overlapping vector bionomics and spatiotemporal distribution, effective and coordinated targeting of the most appropriate vector control interventions will require updating the geographical range of the different diseases and determining their vector species. This will facilitate the integration of vector control interventions and multidisease control approaches aimed at rational and synergistic use of available resources. This also necessitates development of an information system in which data collected is managed by MoH and made available to all beneficiaries [13].

While preventive chemotherapy remains the main stay of reducing the extensive morbidity associated with lymphatic filariasis (LF), onchocerciasis and schistosomiasis, vector control is critical [34]. The body of evidence pointing to the feasibility of multiple vector-borne disease control keeps increasing (Table 3). Vector control has been highly effective in the control of onchocerciasis [35], and might potentially have a significant role in elimination of LF [36]. The current scale-up of LLINs for malaria control may also reduce transmission of LF and visceral leishmaniasis (VL). Recently, IVM has been proposed as the main strategy in areas where LF and loa loa are co-endemic and, where preventive chemotherapy is not feasible, with LLINs being the only intervention option [37]. Considering the relative toxic, expensive and lengthy treatment coupled with the slow development of new drugs and adequate diagnostic tools [38,39], vector control has the potential to effectively reduce the burden of human African trypanosomiasis (HAT) and VL [40-43], but it is rarely implemented because of a lack of financial resources [27].

**Table 3:** Chemical-based and non-chemical vector control methods applicable in South Sudan

**Vector control method**	**Major vectors targeted**	**Vector-borne disease targeted**
Indoor residual spraying	Indoor biting/resting female *Anopheles* mosquitoes; phlebotomine sandflies	Malaria, lymphatic filariasis, visceral leishmaniasis
Long-lasting insecticidal nets	Indoor biting/resting female *Anopheles* mosquitoes; phlebotomine sandflies	Malaria, lymphatic filariasis, visceral leishmaniasis
Other insecticide-impregnated materials	*Anopheles, Aedes, Culex* mosquitoes; phlebotomine sandflies; tsetse flies; *Simulium damnosum* blackflies	Malaria, dengue, lymphatic filariasis, Human African trypanosomiasis, onchocerciasis
Molluscicides	Freshwater snails (*Biomphilaria, Bulinus, Onchomelania*); mosquitoes *Anopheles, Aedes, Culex*	Schistosomiasis, lymphatic filariasis, malaria, dengue
Insect traps	*Anopheles, Aedes, Culex* mosquitoes; tsetse flies	Malaria, dengue, human African trypanosomiasis
Chemical and biological Larvicides	*Anopheles, Aedes, Culex* mosquitoes; *Simulium damnosum* blackflies	Malaria, dengue, lymphatic filariasis, onchocerciasis
Environmental modification/manipulation	*Anopheles, Aedes, Culex* mosquitoes; freshwater snails (*Biomphilaria, Bulinus, Onchomelania*)	Malaria, dengue, lymphatic filariasis, schistosomiasis
House modification	Indoor biting/resting female *Anopheles* mosquitoes	Malaria, lymphatic filariasis
Larvivorous fish	*Anopheles, Aedes, Culex* mosquitoes	Malaria, lymphatic filariasis
Non-larvivorous natural predators	Freshwater snails (*Biomphilaria, Bulinus, Onchomelania*)	Schistosomiasis
Polystyrene beads	Mosquitoes	Malaria, dengue, lymphatic filariasis
Topical repellents	Mosquitoes; tsetse flies	Human African trypanosomiasis

While provision of guidance and technical insight to policymakers and programme managers seeking to prevent, control and eliminate vector-borne diseases are key, overcoming vector-borne diseases requires inter- and intrasectoral collaboration, including community empowerment and participation [7]. This ensures their adequate participation in the planning, design and implementation of vector control interventions [13]. Deployment of effective and sustainable interventions would be ascertained through an appropriate regulatory and legislative framework for public health. This requires adaptation and enforcement of relevant environmental and health policies in the areas of impact assessment, agricultural policies in relation to integrated pest management and financial policies for the exemption of taxes and tariffs of vector control supplies. Effective IVM is based on the strength of intersectoral coordination. Because of the interdependency of different public and private sector organizations in relation to health and to avoid duplication of meager resources, effective coordination mechanisms and inclusion of vector control considerations in partner policies is essential.

The lack of clear career paths for entomologists in national health systems and the inadequate training of programme managers in vector control and sound management of pesticides pose operational challenges and threaten vector control efforts [9]. Capacity building for IVM has to be strengthened in order to exploit the full potential of vector control to interrupt transmission and sustain progress made in control of vector-borne diseases. It is imperative to develop essential physical infrastructure (insectaries, laboratories and capacity for operational research) and strengthen the requisite technical and programme (training in entomology and vector control) or project management skills at national and local levels. Establishment of a vector control unit in the MoH would suffice to set a scene for tighter integration of vector-borne disease control programmes and rigorous coordinated routine surveillance, thus providing mutual benefits and offering more effective protection against a range of different debilitating illnesses.

## Conclusions

The potential for integrating vector-borne disease control is enormous in South Sudan. However, strengthened coordination, intersectoral collaboration and institutional and technical capacity for entomological monitoring and evaluation, including enforcement of appropriate legislation are crucial. Intersectoral collaboration would strengthen decision-making among policymakers, vector-borne disease control programme managers and various other partners. Capacity to manage public health pesticides, coordination with agriculture and adaptation of relevant policies and their enforcement, including planning and delivery of interventions is needed in South Sudan. To maximize the impact of vector control interventions, identification of relevant community perceptions and development and promotion of awareness messages for behavioural change impact is critical. There is need for a sustainable and legally recognized national IVM coordinating body with members drawn from the different sectors, together with establishing a vector control unit in the MoH that addresses all vector-borne diseases with clear career opportunities. This paper re-echoes the need for sustained support and also serves as an archetype for similar environments.

## Abbreviations

EMRO: Eastern Mediterranean Region; HAT: Human African trypanosomiasis; IRS: Indoor residual spraying; IVM: Integrated vector management; LF: Lymphatic filariasis; LLINs: Long-lasting insecticidal nets; MOH: Ministry of health; VL: Visceral leishmaniasis; WHA: World health assembly; WHO: World Health Organization; WHOPES: WHO pesticide evaluation scheme.

## Competing interests

The authors declare that they have no competing interests.

## Authors’ contributions

EC conceived the idea and drafted the manuscript; JMG, MBM, RLL, UH, SB and AM collaborated and critically reviewed the article. All authors read and approved the final manuscript.
